# Antenatal psychosocial risk status and Australian women’s use of primary care and specialist mental health services in the year after birth: a prospective study

**DOI:** 10.1186/s12905-016-0344-0

**Published:** 2016-10-25

**Authors:** Virginia Schmied, Rachel Langdon, Stephen Matthey, Lynn Kemp, Marie-Paule Austin, Maree Johnson

**Affiliations:** 1 School of Nursing & Midwifery, Western Sydney University, Locked Bag 1797 Penrith, Sydney, 2751 NSW Australia; 2 Centre for Applied Nursing Research (a joint facility of the South Western Sydney Local Health District and Western Sydney University, Liverpool, Australia; 3 School of Psychology, University of Sydney and Research Director, Infant, Child & Adolescent Mental Health Service, South West Sydney Local Health District, Sydney, Australia; 4 Chair, Perinatal Mental Health Unit University of New South Wales & St John of God Health Care, Burwood , Sydney, Australia; 5 The Black Dog Institute, Prince of Wales Hospital, Sydney, Australia; 6 Faculty of Health Sciences, Australian Catholic University, North Sydney, NSW Australia; 7 The Ingham Institute for Applied Medical Research, Liverpool, Sydney, NSW Australia

**Keywords:** Perinatal mental health, Health services research, Service utilisation, Mental health services, General practice, Child and family health nursing, Midwifery

## Abstract

**Background:**

Poor mental health in the perinatal period can impact negatively on women, their infants and families. Australian State and Territory governments are investing in routine psychosocial assessment and depression screening with referral to services and support, however, little is known about how well these services are used.

The aim of this paper is to report on the health services used by women for their physical and mental health needs from pregnancy to 12 months after birth and to compare service use for women who have been identified in pregnancy as having moderate-high psychosocial risk with those with low psychosocial risk.

**Methods:**

One hundred and six women were recruited to a prospective longitudinal study with five points of data collection (2–4 weeks after prenatal booking, 36 weeks gestation, 6 weeks postpartum, 6 months postpartum and 12 months postpartum) was undertaken. Data were collected via face-to-face and telephone interviews, relating to psychosocial risk factors, mental health and service use. The prenatal psychosocial risk status of women (data available for 83 of 106 women) was determined using the Antenatal Risk Questionnaire (ANRQ) and was used to compare socio-demographic characteristics and service use of women with ‘low’ and ‘moderate to high’ risk of perinatal mental health problems.

**Results:**

The findings indicate high use of postnatal universal health services (child and family health nurses, general practitioners) by both groups of women, with limited use of specialist mental health services by women identified with moderate to high risk of mental health problems. While almost all respondents indicated that they would seek help for mental health concerns most had a preference to seek help from partners and family before accessing health professionals.

**Conclusion:**

These preliminary data support local and international studies that highlight the poor uptake of specialist services for mental health problems in postnatal women, where this may be required. Further research comparing larger samples of women (with low and psychosocial high risk) are needed to explore the extent of any differences and the reasons why women do not access these specialist services.

## Background

Poor mental health in the perinatal period, defined as from conception to 1 year after birth, is a global problem [1]. Between 10 and 20 % of women report depressive symptoms at some point in pregnancy and or the year after birth [2, 3], yet many are not diagnosed or treated [4, 5]. Perinatal depression and anxiety are associated with poorer outcomes for women including risk of self-harm, poor physical health, breakdown in relationships, unhappiness in the maternal role and, for some, less capacity to nurture their infant [2, 6, 7]. Research shows that the early years of life strongly influence infant development [8–10]. Poorer cognitive functioning, impairments in language, physical, psychosocial, emotional and behavioural problems have been identified in infants of women with perinatal mental health (PMH) problems [11–13].

Key risk factors for poor maternal PMH include: maternal mental health problems (prior and current), physical ill-health, substance misuse, past history or current abuse and situational factors such as quality of significant relationships, lack of support, lower socio-economic status and living in areas of disadvantage [3, 14–16]. Further, the mother’s birth experience, difficulties with breastfeeding or having an infant with health problems, born prematurely or with a difficult temperament [17–20] are also associated with poorer maternal mental health outcomes. Appropriate social support, adequate self-esteem, adequate social and economic circumstances, and maternal attachment state of mind [15, 21–23] are known protective factors. Another potentially significant protective factor is the woman’s propensity to seek help from health and other professionals [24].

Studies of therapeutic and support intervention indicate that PMH problems can be minimised if women engage in appropriate services [25]. Significant efforts have occurred both in Australia and internationally to redesign and strengthen services, and to provide coordinated and tiered care [1]. The Australian health service system includes publicly funded universal services (e.g. prenatal care provided by midwives, child and family health nursing services for families with children 0 to 5 years of age) located within primary care; targeted support for children and families with additional needs (e.g. sustained nurse home visiting programs) [26–28]; secondary and tertiary services for those with higher levels of need including mental health, drug and alcohol services, co-ordinated team management with multi-component interventions [29–31] offering evidence based interventions. To identify women and families who will benefit from early intervention and treatment, many countries including Australia are investing in routine (universal) psychosocial assessment and depression screening for pregnant women and new mothers [1, 32, 33]. In New South Wales (NSW), Australia, a new policy platform for maternal and child health has been implemented and includes routine psychosocial assessment and depression screening and a coordinated service structure. Such assessment and screening was trialled in a number of health districts from 2000 and formally implemented as policy in 2009 [34]. Three levels of risk are identified through the psychosocial assessment and depression screening, t: Level 1: no vulnerabilities detected and women receive universal midwifery and primary care services; Level 2: women may be experiencing one or more issues such as previous history of postnatal depression or mental health concerns; an unemployed partner, death of a person close to her in the past year, unstable housing, and lack of social support or poor partner relationship; and Level 3: complex risk factors, such as current maternal mental illness, substance use, or domestic violence or previous child protection involvement. Women identified with multiple Level 2 or any Level 3 risk factors are referred to a multidisciplinary case meeting, where referral to more specialised services, for example, drug and alcohol treatment, is determined [35]. Some health areas have discreet specialist perinatal and infant mental health (PIMH) services. These multidisciplinary teams provide individualised care for women and their families who are identified as at risk for poor perinatal mental health outcomes [31]. Myors et al. [31] examined the range of interventions that specialised perinatal and infant mental health clinicians used in their work with women and infants. Primarily the interventions delivered depended upon the training of the clinician, such as family of origin work/genograms, non-directive counselling, and strategies to manage anxiety and depression. Clinicians also emphasised the therapeutic relationship and the interventions they use within an attachment-based framework.

However, many women identified with risk factors for, or identified as having a diagnosis of postnatal depression, do not take up services [5, 36]. Factors known to influence service uptake include current mental health, symptom awareness, normalisation of postpartum depression, timeliness of care, perception of usefulness of service, service quality and perceived stigma of being labelled as ‘depressed’ [4, 24, 37, 38]. Factors such as help seeking behaviour may potentially mediate the relationship between risk factors and outcomes [39, 40].

The aim of this paper is to report the health services used by women for their physical and mental health needs from pregnancy to 12 months after birth and to compare service use for women who have been identified in pregnancy of having ‘psychosocial risk’.

## Methods

### Study design

This study employed a prospective longitudinal design with repeated measures following women from pregnancy to 12 months postpartum. Demographic data were collected when the woman consented to participate in the prenatal clinic immediately prior to her first prenatal appointment. Data relating to risk factors including mental health and service use were collected at 5 points in time: 2–4 weeks after prenatal booking, 36 weeks gestation, 6 weeks postpartum, 6 months postpartum and 12 months postpartum.

### Setting

The study was conducted at two metropolitan public hospitals in NSW Australia with a combined birth rate in 2012 of just over 8,000. Both have a well-established process of assessment and screening and a referral pathway and network of services developed over the past 10 years. Although state-wide policy and guidelines [35] recommend the services and professionals required to form the service network, these will vary across the local health districts and the individual mental health and maternity and child health services. Thus the pathways, services and staffing differed in each district depending on resources and access to specialised staff. In site 1 numerous pathways were available for women following assessment in the antenatal clinic. These included a sustained nurse home visiting program; specialised perinatal and infant mental health service with four staff working as therapists/counsellors and one psychiatrist. In addition, drug and alcohol services and adult mental health services were available. In site 2 a perinatal coordinator, who was a mental health nurse, was employed to follow up with clients following referral. Some women were referred to antenatal group for anxiety concerns; others received care coordinated by a social worker, mental health nurse, psychologist and women had access to a specialist perinatal psychiatrist. Drug and alcohol services and adult mental health services were also available.

### Sample

Pregnant women booked to give birth at either site were recruited to the study between October 2010 and November 2010. Inclusion criteria were nulliparous and multiparous women ≥ 12 weeks gestation at first prenatal visit, who had public health care insurance (Medicare), age ≥18, and sufficient proficiency in English to participate in structured interviews. The only exclusion criteria were women who required an interpreter for the prenatal appointment.

### Recruitment

An information letter about the study was included with the confirmation letter for the first hospital appointment. A research assistant approached women in the clinic prior to their first appointment, provided them with further information about the study, and invited them to participate. If women agreed to participate, they completed formal written consent and demographic information including; age, parity, relationship status, country of birth, language spoken at home, employment status. All participants were offered a small remuneration for participation, comprising a $A25 Coles/Myer gift voucher, given after the first and third interviews.

In the study recruitment period, a total of 700 women attended their first antenatal booking visit at the study sites. Of these approximately 20 % did not meet the study criteria. Of the eligible women, 125 (22 %) were invited to participate by the research assistant who was only available 2 days of the week. One hundred and six women (85 %) were recruited to the study and completed consent forms and some demographic details at recruitment. Fifteen percent declined to participate for a range of reasons including they did not have time, did not speak sufficient English or they were not interested.

### Data collection

Data were collected using structured face-to-face and telephone interviews using validated instruments. The first interview (T1) occurred 2–4 weeks after the prenatal appointment and face-to-face interviews occurred either in the clinic or in the woman’s home. Some women (40 %) preferred a telephone interview at this time. All interviews at 36 weeks gestation (T2) were by phone and 60 % of interviews at 6 weeks (T3) were by phone. All interviews at 6 (T4) and 12 months (T5) were by phone. Interviews at T1, T3 and T5 took between 20 and 40 min and the interviews at T2 and T4 were between 10 and 15 min long.

### Study measures

The instruments selected for this study have been well-validated and most have previously or are currently being used in Australian studies. Table 1 outlines data collection and measure at each time point.

**Table 1:** Data collection points and study measures

Time	Measures	Instrument + validation studies + indications of previous use
Maternal measures of perinatal mental health, psychosocial risk and mediating factors		
T1, T3 T4, T5	Mental Heath	Edinburgh Postnatal Depression Scale (EPDS) [41] Hospital Anxiety and Depression (HADS) anxiety subscale [42]
T1	Psychosocial risk	Antenatal Risk Questionnaire (ANRQ)
T1	Help-seeking	General Help-Seeking Questionnaire [43]
Service need and utilisation		
T1-T5	Maternal rating of need, service utilisation and service quality	Emotional Health Service Utilisation Questionnaire Comprises: 1) services the woman accessed for help; 2) types of health professionals seen; 3) types of treatments received; 4) when health professionals seen (pregnancy or postpartum); 5) rating of the helpfulness of contacts/treatments.

*Maternal mental health* was measured at T1, T3, T4, T5 using the Edinburgh Postnatal Depression Scale: (EPDS). This is a 10 item self-report measure designed to screen women for symptoms of emotional distress during pregnancy and the postnatal period [41]. The Hospital Anxiety and Depression Scale (anxiety subscale) (HADS-A), a 7 item scale, was used at T1, T3, T4, T5 to assess symptoms of anxiety [42].

*Psychosocial risk status* was determined using the Antenatal Risk Questionnaire (ANRQ) [32] together with domestic violence screening and questions around drug and alcohol use at T1 (see Table 2). The ANRQ consists of 12 items that assess the following domains: emotional support from mother in childhood, past or current depressed mood or mental illness and treatment received, perceived level of support available following the birth of the baby, partner emotional support, life stresses in the previous 12 months, personality style (anxious or perfectionist traits) and history of abuse (emotional, physical and sexual) [32]. It is scored using a combination of categorical and continuous data, with a possible maximum score of 62 and minimum score of 5 [32]. A score of 23 or more or the presence of any of the weighted critical factors (a history of depression, psychiatric diagnosis and/or abuse or emotional neglect in childhood) will identify women in the moderate to high risk group in this sample [32].

**Table 2:** Examples of the additional questions to the ANRQ

Series of questions on household smoking	
Alcohol and other drugs	
On average, how many days a week do you drink alcohol?	
Do you consider alcohol to be a problem in your household?	
Do you consider drugs to be a problem in your household?	
Domestic Violence questions	
Within the last year have you been hit, slapped, or hurt in other ways by your partner or ex-partner?	
Yes □	
No □	
Are you frightened of your partner or ex-partner?	
Yes □	
No □	
(With a series of sub questions if the woman answers yes).	

*Help seeking behaviour* was measured at T1 using the General Help-Seeking Behaviour (GHSB) questionnaire [43] developed to formally assess two aspects of help-seeking: 1) current intentions to seek help from different sources for different problems; and 2) quantity and quality of previous professional psychological helping episodes.

#### Maternal rating of need, service utilisation and service quality

Four components were assessed at T2, T3, T4, T5 based on a previous survey by Reay et al. [5] and included: 1) services accessed from a structured list which included: midwifery, obstetric, general practitioner, other specialists e.g. endocrinologist and child and family health (CFH) nursing services, allied and mental health services, family support services, drug and alcohol services, telephone helpline and peer support eg Australian Breastfeeding Association; 2) types of treatments received; 3) time points when service provided and, 4) rating of the helpfulness of service on a scale from 1 to 5 where 1 is very helpful and 5 is very unhelpful.

### Data analysis

Data were analysed using IBM SPSS Statistics for Windows, Version 20.0 [44]. The prenatal risk status of women was calculated and formed the basis for comparisons. Chi-square statistics and t-tests were calculated to determine differences in health service use for women at moderate/high risk compared to women at low risk. Given that statistical significance is affected by sample size [45], effect size statistics (ES) have also been calculated to report on whether any statistically significant differences are in fact clinically meaningful, or if there is an indication from the ES measure that a non-statistically significant result is likely due to the small sample size. For chi square analyses, phi (ϕ) values of 0.15 or more are considered clinically meaningful, while for t tests d values of 0.3 or more are considered clinically meaningful [45, 46].

## Results

At Time 1, 83 women completed the surveys, including demographic data. Numbers of women completing the surveys varied across the different time points, with 66 at Time 3, 50 at Time 4 and 53 at Time 5.

### Demographic characteristics and risk status

The risk status of women was calculated using the ANRQ at T1. Eighty-three women completed this survey, of whom 33 (39.8 %) were classified as being at moderate to high risk for poor perinatal mental health outcomes, that is, either scoring 23 or more on the ANRQ or having any of the weighted risk factors such as childhood sexual abuse.

No demographic differences were found between women identified as moderate/high risk and those who were low risk (see Table 3). Just over half of the women surveyed were born outside Australia, though the majority of women spoke English at home (see Table 3). The majority of women had a partner, and the mean age was 30.32 years. More than half the women had completed a university degree. While not statistically significant, more women who were at moderate/high risk reported they were unemployed compared to those at low risk (21.9 % vs 10.4 %; ϕ = 0.16).

**Table 3:** Characteristics of women at high compared to those at low risk (ANRQ)

Characteristics	ANRQ risk status		
	Moderate/high risk	Low risk	χ^2^ (sig)
	N (%)	N (%)	
Country of birth			
Australia	14 (43.8)	21 (42.9)	.006 (.937)
Other	18 (56.3)	28 (57.1)	
Language spoken at home			
English	24 (75.0)	37 (77.1)	.046 (.830)
Other	8 (25.0)	11 (22.9)	
Education level			
Completed Year 10 or equivalent	2 (6.3)	0 (0.0)	9.074 (.059)
Completed Year 12 or equivalent	5 (15.6)	6 (12.2)	
Trade apprenticeship/TAFE qualification	5 (15.6)	13 (26.5)	
University degree	17 (53.1)	30 (61.2)	
Other	3 (9.4)	0 (0.0)	
Marital status			
Partnered	30 (93.8)	49 (100)	3.140 (.076)
Unpartnered	2 (6.3)	0 (0.0)	
Employment status			
Employed (full time or part time)	17 (53.1)	31 (64.6)	4.988 (.289)
Full time mother/home duties	4 (12.5)	10 (20.8)	
Student (either employed or unemployed)	3 (9.4)	1 (2.1)	
Unemployed	7 (21.9)	5 (10.4)	
Other	1 (3.1)	1 (2.1)	
Pregnancy planning			
Planned to get pregnant around about now	15 (45.5)	27 (54.0)	3.209 (.360)
Planned to get pregnant but not so soon	9 (27.3)	17 (34.0)	
Unintended	7 (21.2)	5 (10.0)	
Other	2 (6.1)	1 (2.0)	
Parity			
Primip	15 (50.0)	21 (44.7)	.208 (.648)
Multip	15 (50.0)	26 (55.3)	
Delivery type			
Normal vaginal delivery	15 (71.4)	24 (63.2)	3.030 (.220)
Caesarean section	3 (14.3)	12 (31.6)	
Vacuum extraction	3 (14.3)	2 (5.3)	
Problems with delivery			
0 to 2	11 (47.8)	16 (39.0)	.468 (.494)
3 or more	12 (52.2)	25 (61.0)	

Approximately half the women surveyed were pregnant for the first. While not statistically significant, a higher proportion of women at moderate/high risk reported their pregnancy as being unplanned compared to those women at low risk (21.2 % vs 10.0 %; ϕ = 0.16). There were no differences in type of birth experienced (normal vaginal birth vs elective or emergency caesarean section) or postnatal problems related to delivery (see Table 3).

Women also completed the EPDS and the HADS anxiety scale at Time1. As would be expected, given that the ANRQ includes current mood as a risk, moderate/high risk women were statistically and clinically significantly more likely than low risk women to have higher scores on both the EPDS (t = −2.672, *p* = .009) and the HADS anxiety Scale (t = −2.483, *p* = .015) (see Table 4).

**Table 4:** Mental health characteristics of women at high compared to those at low risk (ANRQ)

Characteristics	ANRQ risk status		
	Moderate/high risk	Low risk	χ^2^ (sig)
	N (%)	N (%)	
HADS status			
< 11	25 (75.8)	44 (89.8)	2.913 (.088)
≥ 11	8 (24.2)	5 (10.2)	
EPDS status			
< 13	26 (78.8)	47 (95.9)	5.922 (.015)
≥ 13	7 (21.2)	2 (4.1)	
	Mean (SD)	Mean (SD)	t (sig)
HADS (total)	8.91 (2.95)	7.61 (1.78)	−2.483 (.015)
EPDS (total)	6.85 (6.28)	3.84 (3.94)	−2.672 (.009)
Confidence as a person	7.48 (1.33)	7.52 (1.68)	101 (.920)
General help seeking behaviour	42.45 (9.16)	44.88 (8.49)	1.234 (.221)

Women were also asked to rate their personal confidence and their general help seeking behaviour. On these two scales, moderate/high risk women scored similarly to low risk women (see Table 4).

### Willingness to seek help

The women were asked how likely they were to seek help in relation to emotional issues. Very few women in either risk group indicated they were unlikely to seek help from anyone (moderate/high risk = 6.7 %; low risk = 4.3 %). Women, both those at moderate/high risk and those at low risk, said that they were most likely to seek help from their intimate partner (90.6 % and 98.0 % respectively). Similar proportions of women in both risk groups reported being likely to seek help from a friend moderate/high risk = 81.2 %, low risk =71.4 %), parent (moderate/high risk = 72.7 %, low risk =80.0 %), another family member (moderate/high = 51.5 %, low = 68.8 %), or other relatives (moderate/high = 39.4 %, low = 53.1 %). Almost half the women in both groups reported being willing to seek help from a mental health professional (moderate/high = 54.5 %, low = 44.0 %), or a GP (moderate/high = 36.4 %, low = 46.0 %). Relatively few women in both risk groups reported being willing to seek help from a help line, high risk (18.2 %) and low risk (22.4 %).

### Use of services

Following on from asking about their willingness to seek help from various services, women self-reported service use (see Table 5). Very few women, either moderate/high risk or low risk, reported accessing mental health services at any of the postnatal survey times. The most commonly accessed mental health service was a telephone helpline (see Table 5).

**Table 5:** Number of women who have used services at least once in the 12 months following birth

Service	PN 6 week		PN 6 months		PN 12 months	
	Mod/high risk (*n* = 25)	Low risk (*n* = 41)	Mod/high risk (*n* = 17)	Low risk (*n* = 33)	Mod/high risk (*n* = 19)	Low risk (*n* = 34)
Mental health						
Telephone helpline	4 (16.0)	5 (12.2)	2 (11.8)	7 (21.2)	1 (5.3)	7 (20.6)
Psychiatrist	1 (4.0)	0 (0.0)	1 (5.9)	1 (3.0)	1 (5.3)	2 (5.9)
Social worker	1 (4.0)	1 (2.4)	1 (5.9)	1 (3.0)	1 (5.3)	0 (0.0)
Counsellor	0 (0.0)	1 (2.4)	1 (5.9)	1 (3.0)	2 (10.5)	3 (8.8)
Universal health services						
Midwife	13 (52.0)	19 (46.3)	-	-	-	-
GP (for self or baby)	15 (60.0)	30 (73.2)	-	-	-	-
GP (for self)	-	-	14 (82.4)	31 (93.9)	14 (73.7)	28 (82.4)
GP (for baby)	-	-	16 (94.1)	32 (97.0)	18 (94.7)	34 (100)
CFH nurse (at clinic)	12 (48.0)	17 (41.5)	11 (64.7)	19 (57.6)	8 (42.1)	10 (29.4)
CFH nurse (at home)	15 (60.0)	19 (46.3)	8 (47.1)	14 (42.4)	0 (0.0)	2 (5.9)
Specialist health services						
Specialist (for self or baby)	7 (28.0)	11 (26.8)	7 (41.2)	14 (42.4)	-	-
Obstetrician	2 (8.0)	5 (12.2)	-	-	-	-
Specialist (for self)	-	-	-	-	1 (5.3)	6 (17.6)
Specialist (for baby)	-	-	-	-	2 (10.5)	7 (20.6)
Lactation consultant	4 (16.0)	9 (22.0)	1 (5.9)	6 (18.2)	0 (0.0)	0 (0.0)
Other						
Emergency department	1 (4.0)	6 (14.6)	3 (17.6)	2 (6.1)	8 (42.1)	5 (14.7)^a^

^a^X^2^ = 4.943, *p* = .026

Women frequently used universal health services at the three postnatal survey times, with no statistically significant differences between the two groups of women at each of the survey times. The majority of women at each postnatal survey time visited a GP at least once in the first postnatal year either for themselves or their baby (moderate/high risk ranging from 60 to 94.7 % and low risk ranging from 73.2 to 100 %). Similarly, a large proportion of women also visited a child and family health nurse during the postnatal period.

The use of specialist (e.g. obstetric, endocrinology or paediatrics but non mental health) services, either for themselves or their baby, was also common during the postnatal period, though no statistically significant differences were found in the use of these services between moderate/high risk and low risk women, and specialist mental health service use was very low.

Clinically significant differences were found for the two risk groups attending the emergency department at all three time points (see Table 5). Low risk women were more likely than mod/high risk women to attend this service at 6 weeks (ϕ = 0.17), while the opposite was found for at 6 month time point (ϕ = 0.18). Statistical and clinical significance was found at 12 months, with more mod-high risk women attending the emergency department than low risk women (42.1 % vs 14.7 %, χ^2^ = 4.943, *p* = .026; ϕ = .31).

Women were also asked if they had accessed or attended any other kind of service. As these services were not specifically prompted for, and the number of women who responded was small, no significance testing was undertaken. Women nominated various services, the most common being those for parenting support (e.g. child and family health nursing services). A relatively high proportion of women reported accessing general parenting support services at each of the survey times (6 weeks: moderate/high risk = 48 %, low risk = 24.4 %; 6 months: moderate/high risk = 47.1 %, low risk = 54.5 %; 12 months: moderate/high risk = 63.2 %, low risk = 61.8 %). Other parenting support services accessed included facilitated groups or peer support groups for PND.

### Use of mental health services

**Fig. 1:**
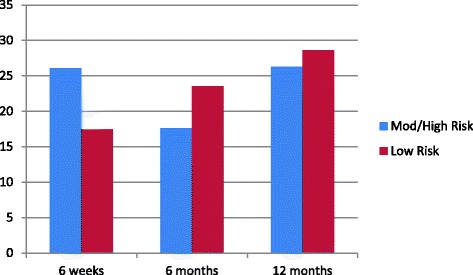
Percent of women using mental health services

Only a small number of women accessed mental health services and there were no statistically or clinically significant differences between the number of women reporting accessing mental health services at any of the postnatal survey times in the two risk categories. Figure 1 below demonstrates the percent of women accessing services for mental health (including psychiatrist, psychologist/counsellor, telephone helpline) at the time of the 6 week, 6 month and 12 month postnatal surveys.

## Discussion

Almost forty percent of women in this study were identified as being moderate/high risk for PMH problems. This is similar to previous reports of risk status in these sites [47]. Over half of the sample was born overseas and 25 % spoke a language other than English at home. This reflects the population of the study sites; one site was in an area of known socio-economic disadvantage with a high proportion of migrants from non-English speaking backgrounds and the other site in an area with a high proportion of English speaking migrants. Living away from family and lack of social support is a known stressor or risk factor in the perinatal period [3, 48]. Studies in Australia and internationally consistently report higher rates of PMH problems among migrant women [49–51] and a recent study of women living in the same geographical area confirmed this pattern [15].

Importantly, few women reported that they were unlikely to seek help from anyone for emotional issues. However, the majority indicated that they were most likely to seek help from their partners, their family or friends and a lower proportion of women in both the high risk and the low risk group reported they would use a GP or mental health professionals. These views on help seeking are confirmed by studies reporting behaviour or actions by women when referred to services [52, 53]. Henshaw et al. [53] reported 83 % of women consulted friends and family about symptoms of perinatal depression while 50 % consulted health care professionals. Reilly et al. in an Australian survey of 590 women experiencing significant emotional distress perinatally, reported that 30.3 % women saw a mental health professional; 42.6 % a GP; while 90.3 % sought help from their partner, family or social network [54]. In a qualitative study Abrams et al. (2009) found that women overwhelmingly recommended self-help practices such as “talking it out” with other mothers in lieu of formal mental health care.

### Professional help – primary care/universal services

Less than half of the women indicated on the GHSB questionnaire that they were willing to seek help from their GP for emotional health issues. Yet almost all women (both mod/high and low risk groups) visited their GP at least once from birth to 12 months for both themselves and their baby. We do not know however whether women were asked about or discussed mental health issues in visits to the GP. This high use of universal and primary care services is supported by other Australian studies [51, 55]. Lansakara et al. [56] reported that 97 % of women had contact with a primary health care practitioner, either GP or CFH nurse with regard to their own health at least once during the first three months postpartum, and most (approximately 75 %), contacted both of these professionals at least once in first three months. Farr et al. [57] also reported that infants of mothers with perinatal depression or anxiety were as likely to attend well baby visits and receive immunisations as their counterparts. This reinforces the importance of generalist health professionals and those providing universal CFH services taking the opportunity to inquire sensitively about the social and emotional health of pregnant women and parents with young infants.

Women identified as moderate/high risk were also statistically significantly and clinically more likely than women with low risk to report attending the emergency department at the 12 month. We do not know if this was to access care for themselves or their baby. It is possible that women may present at ED with physical health problems or complaints and that this may be a proxy for emotional distress or lack of support. They may be less likely to have a regular GP and use ED instead. Farr et al. [57] also found that infants of mothers with prenatal and postpartum depression or anxiety were more likely to have ≥6 sick/emergency visits than infants whose mothers did not have PMH problems. Chee et al. [58] similarly reported that women who had brought their infants for three or more non-routine visits to the infant’s doctor had a significantly higher prevalence of depression. They were also more likely to have poorer perceived emotional support from their families.

### Use of mental health services

Approximately half of the women surveyed reported they were willing to seek help from a mental health professional. In reality few women, either high risk or low risk, reported accessing mental health services at any of the postnatal survey times. At the time of the 12 month postnatal survey, approximately 26 % of women at mod/high risk reported accessing any services for mental health and primarily this was a telephone helpline service. This apparent low uptake of mental health services by women considered to be at risk for poor mental health outcomes is reported internationally. In the US, one study reported that only 34 % of women with probable perinatal depression utilised professional help [52], while another reported a 50 % uptake [53]. Smith et al. studied a group of pregnant and postpartum women and found that of those referred for additional services for mental health concerns, 38 % attended at least one mental health visit, while only 6 % remained in treatment during the entire 6-month follow-up interval. They found however that postpartum women were more likely than pregnant women to attend for mental health treatment. Importantly Smith et al. [59] found that women who received a referral to services at the same site as their pregnancy and or postpartum care were more likely to attend a mental health appointment.

Recent Australian studies demonstrated higher rates of uptake for women experiencing postnatal depression, Reay et al. [5] reported 63 % of women with a diagnosis of depression accepted treatment and Lansakara et al. [51] reported 65.5 % of women experiencing depressive symptoms but only 44 % of women experiencing anxiety symptoms alone had spoken to a health professional, about their concerns. Reilly et al. [54] found 62.6 % women experiencing any mental health symptoms in the postnatal period sought formal health assistance (from any source). This does not mean they necessarily remained engaged with services.

It appears there may also be some services that are more acceptable to women in the perinatal period. As noted earlier, some LHDs in NSW have established perinatal infant mental health services (PIMHS). To date, there have been few studies reporting the process and outcomes from these services. Myors et al. [40] conducted a mixed methods study to investigate women’s engagement in and perceptions of the PIMH service. They found 71.3 % women referred engaged with the PIMH service. Building trust and therapeutic engagement were central to the work of the PIMH staff and client’s described this as their special time. Furthermore, while women in our study did not report using tertiary level residential parenting services, others have previously found that demand for these unique Australian services (e.g. Tresillian and Karitane in NSW) is high [60]. Christl et al. [61] have recently shown that a high proportion of women using both residential and outpatient parenting services in the first postnatal year have high scores on the ANRQ (postnatal version).

Contrary to what women reported on the GHSB questionnaire, the most commonly accessed mental health service was a telephone helpline and the use of helplines was slightly higher for women with high risk at 6 weeks. At the 6 month and 12 month postnatal survey times these variations were reversed, with fewer high risk women reporting using a telephone helpline compared to low risk women.

### Barriers to help seeking

The low use of mental health services in our study may have occurred for a range of reasons. First, women who reported previous mental health issues or childhood abuse may have had support to address these issues and consequently did not believe they needed any further services. In a qualitative study of a sub sample of these study participants, some women indicated to midwives conducting psychosocial assessment they had ‘dealt’ with these concerns [62].

Stigma associated with mental health issues and feelings of shame, embarrassment, fear of being labelled and lack of understanding and/or support from family are well-known to impede help-seeking [37, 59, 63–66]. This is accentuated by society’s view on the nurturing role of the ‘mother’ and women report feeling pressure to be ‘a good mother’, hiding their negative feelings because they do not want to be seen as not being able to cope [67]. This appears to be particularly so for women from non-English speaking backgrounds [49, 63, 68]. Some women fear that coming forward with what they are experiencing may put them or their partners’ legal status in the country at risk or they at risk of losing their infant [4, 69].

The lack of knowledge of perinatal depression means women may not recognise the associated symptoms, leading to belief in myths and/or somatisation whereby these symptoms are dismissed as a normal part of motherhood [64, 65, 70]. Cultural belief systems and language barriers also hinder women’s help-seeking behaviours [37, 63, 66, 71]. The denial or dismissal of mental health problems is reinforced by health services that do not respond sensitively to the emotional needs of women and for example, in pregnancy maintain focus on the fetus, dismissing the mother and her needs [64, 65, 72, 73]. Many women may not seek help due to their lack of time, child care and experience of transportation issues [37, 63, 64, 66, 70, 72]. It is important to re-emphasise however that being ‘at risk’ for perinatal mental health problems does not mean that a woman will develop a problem/s. In this study, we also do not know what other social supports women may have been using, although some did indicate in open text responses that they talked with family and friends about their concerns.

Lack of service use may also be the result of service availability and accessibility. There are many reports, both in Australia and internationally, of the limited access to primary, secondary and targeted support services for women with perinatal mental health problems [27, 74, 75] Access issues include that women may not be aware of the services; the services may not be in a convenient location or time or accessible by public transport and women may not have access to child care. The services may also not meet women’s needs.

### Facilitating health service use

Importantly there are also factors known to facilitate service use, such as previous experiences particularly the nature of their relationship with their health care professionals [4, 36]. Knowledge about perinatal depression and positive previous experiences are associated with intention to seek counselling and anticipation of better results [76]. Buist et al. 2007 reported that women who had participated in a trial screening program were better able to recognise depression in a hypothetical case, and also assess their own mental state more appropriately. A recent Canadian general population survey of 1207 respondents also suggests that knowledge about periantal depression is high [77]. They found that 72.7 % of all respondents were aware of the risk for postnatal mental health issues and agreed that women should have universal mental health screening at this time. They also reported that assistance if needed, would best be sought from a GP in the first instance, or alternatively from the woman’s partner.

Prenatal appointments with a midwife or GP and visits to the GP for physical health problems following birth offer an important opportunity to ask women about their mental health. Woolhouse et al. [78] recently reported that women who experienced five or more health problems in the first 3 months after birth had a six-fold increase in likelihood of reporting concurrent depressive symptoms at three months postpartum and a three-fold increase in likelihood of reporting subsequent depressive symptoms at 6–12 months postpartum.

Peer support, either those who have previously experienced PMH problems or who are currently experiencing problems can also promote help seeking behaviours for women, alongside one another [73, 79]. Women have indicated that they find comfort in knowing that they are not alone and report finding women’s stories in magazines and conversations on internet chat sites helpful [73]. A recent study in the US reported that women wanted access to a greater range of different kinds of support for mental health problems, such as educational groups or peer support [80]. They also described significant social and economic needs which were rarely addressed in current services where the biomedical model dominates.

### Training and support for professionals

Universal or primary care service providers such as midwives, CFH nurses and GPs are the first point of contact for women in the prenatal and postnatal periods and currently in NSW, are charged with conducting psychosocial assessment and depression screening. It is important to consider how well midwives, CFH nurses and GPs conduct the psychosocial assessment. A number of women in the nested study by Rollans et al. [62] reported that they were surprised by the questions they were asked at this first meeting with the health professional. Some indicated that they would not answer truthfully if they did have concerns and others who did disclose declined the offer of referral to the multidisciplinary team. A high level of skill and confidence is required to be effective in this role.

### Limitations

The small sample size of the pilot study has not allowed for adequate testing of hypotheses, in particular any indirect relationships between risk status, service use and outcomes. As noted in the methods section, we excluded women who had limited English and it is also likely that some of the women who did not complete the surveys at T1 after recruitment did not speak sufficient English. Therefore the sample may not be representative of the population giving birth at the two study sites, particularly site 1. The participants were also a highly educated group and may not represent the population, again particularly at site 1. The findings from this small study indicate the need to systematically investigate the impact of psychosocial assessment and risk identification on patterns of service use and the subsequent outcomes for women and infants.

## Conclusion

These preliminary data add some support to local and international studies that highlight the limited uptake of specialist services for mental health problems where this may be required. Further research comparing larger samples of women (with low and high risk) are required to explore the extent of any differences and the reasons why women do or do not access these important specialist services. At the same time, research is needed into the service system, to map current pathways and available services as well as ongoing service need and training and support for staff. It is important to remember that being at risk for poor perinatal mental health does not mean that an individual woman will develop problems in the perinatal period or beyond. Specialist perinatal mental health services are important but are limited in the current climate of health service funding and many women may benefit more from being linked to quality primary care services. Close examination of the service use patterns particularly the use of GPs and CFH nurses for mental health support is also needed.

## Abbreviations

ANRQ: Antenatal Risk Questionnaire
CFH nurse: Child and family health nurse
EPDS: Edinburgh Postnatal Depression Scale
GHSB: General Help-Seeking Behaviour
GP: General practitioner
HADS: Hospital Anxiety and Depression Scale
PMH: Perinatal mental health
